# How ants use quorum sensing to estimate the average quality of a fluctuating resource

**DOI:** 10.1038/srep11890

**Published:** 2015-07-08

**Authors:** Nigel R. Franks, Jonathan P. Stuttard, Carolina Doran, Julian C. Esposito, Maximillian C. Master, Ana B. Sendova-Franks, Naoki Masuda, Nicholas F. Britton

**Affiliations:** 1 School of Biological Sciences, University of Bristol; 2 Champalimaud Neuroscience Programme, Champalimaud Centre for the Unknown, Lisbon, Portugal; 3 Department of Engineering Design and Mathematics, UWE, Bristol; 4 Department of Engineering Mathematics, University of Bristol; 5 Department of Mathematical Sciences & Centre for Mathematical Biology, University of Bath.

## Abstract

We show that one of the advantages of quorum-based decision-making is an ability to estimate the average value of a resource that fluctuates in quality. By using a quorum threshold, namely the number of ants within a new nest site, to determine their choice, the ants are in effect voting with their feet. Our results show that such quorum sensing is compatible with homogenization theory such that the average value of a new nest site is determined by ants accumulating within it when the nest site is of high quality and leaving when it is poor. Hence, the ants can estimate a surprisingly accurate running average quality of a complex resource through the use of extraordinarily simple procedures.

Decision-makers interrogating alternative options may have to gather information that is either intrinsically noisy (i.e. the same option varies in the rewards it provides from one moment to the next) or whose completeness, and hence quality, is time dependent. The former may arise when pay-offs vary stochastically such as in so called multi-armed bandit problems^1^,^2^. In this case, one might wish to estimate the average success rate provided by different alternatives that offer rewards that fluctuate wildly^1^,^2^. Speed vs. accuracy trade-offs may also occur when the alternatives are constant because discriminating among them accurately can depend on gathering sufficient information and that takes time. Such speed vs accuracy trade-offs are wide-spread^3^,^4^,^5^,^6^,^7^,^8^,^9^,^10^. Recently, it has been recognized that there may be other trade-offs in decision-making; in addition to speed-accuracy trade-offs, there can, for example, be speed-vs-cohesion (or speed-vs-precision) trade-offs^11^ and speed-vs-value trade-offs^12^.

Collective decision-makers will face a different challenge if some members of a group rate the same alternative as excellent (i.e. of great value) and some rate it as poor. Indeed, economic markets are often governed by collective decision-making where the opinions of others influence how goods are valued^13^. To give a quotidian example, when deciding whether to purchase on-line goods, one might look at the star-rating provided by previous consumers. Consider a case in which 100 reviewers, from a total of 200, rated item **A** as 5 stars whereas the other 100 reviewers gave the same item a rating of 1 star. The average is 3 stars; but not a single person gave item **A** 3 stars. The average for this first item **A** seems almost meaningless. Indeed, one might struggle to evaluate it against an ostensibly similar and identically priced item **B** that all 200 reviewers rated as 3 stars. Which item one chooses, **A** or **B**, might well depend on one’s propensity to gamble.

Furthermore, when swamped with choice, a single poor rating amongst large numbers of excellent ones may cause aversion. This kind of risk aversion, over-reacting to potential losses, is grist to the mill for prospect theory^14^,^15^.

In short, modern consumerism is often imbued with certain elements of collective decision-making and potentially conflicting information. Of course it could be argued that hedonism might play a role in human consumerism whereas ants, for example, should be selected to make value-based decisions^12^. Here, we report a study in which ant colonies make collective decisions between two alternative nest sites. One new nest site is constant and mediocre (metaphorically equivalent to a constant and uniform 3 star rating) and the other fluctuates, for different time periods, between being very good (e.g. a 5 star rating) and poor (e.g. a 1 star rating).

Evaluating a resource that fluctuates in quality raises issues such as how frequently the resource is analysed. A common practice for understanding such systems is to replace spatial or temporal heterogeneity with averaged values. Mathematically this approach falls under the scope of homogenization theory which posits that under appropriate conditions one can safely average out fluctuations within a system before calculating its solutions^16^. In this context, we test whether the implications of this theory hold true by comparing responses of ants to a temporally fluctuating nest and to a constant nest whose quality averages temporally the fluctuating nest.

Our model system is the rock ant (*Temnothorax albipennis*) whose collective decision-making over new nest sites has been investigated extensively^7^,^8^,^17^,^18^,^19^,^20^,^21^,^22^. For studies of house-hunting in other *Temnothorax* species see, for example, Sasaki *et al.* (2013)^23^, Sasaki & Pratt (2013)^24^. *T. albipennis* ant colonies will move to better nest sites even if their current nest site remains the same^25^. Indeed, it seems that a small minority of the workers in each colony are constantly on the lookout for better nest sites^19^. When a worker finds a potential nest site she investigates it thoroughly and weights many criteria^18^. If the nest site is potentially worth further consideration other workers can be recruited to it in a one-by-one fashion through tandem running^21^. If enough workers deem the new nest site of sufficient value their presence there will contribute to a quorum threshold which when surpassed changes the recruitment behaviour from slow tandem running to fast social carrying. The quorum threshold is based on the abundance of ants in the new nest site and it seems to take an ant about 2 min to determine if a quorum has been achieved within a particular nest site^26^. The quorum threshold employed can change in response to environmental conditions and the need for urgency^7^. The tandem running that is used to build towards a quorum is extremely slow^27^. The social carrying that begins at the attainment of a quorum threshold, is fast: it is three times faster than tandem running and signals the start of an emigration and hence the choice of, and commitment to, a new nest site^21^. However, quorums can occur without tandem running when sufficient ants independently find and investigate a new nest site. Quorum attainment without tandem running is common when new nest sites are close to the old nest site^28^ as in the experiments described here. When quorum thresholds are achieved in the absence of tandem running they occur in the absence of positive feedback. These ants check for quorum thresholds by measuring their encounter rates with nestmates in the new nest site^29^, a process which alone must delay their departure. Quorum thresholds are not only flexible, being higher in benign rather than harsh conditions, but they are also based on the ants almost literally voting with their feet^7^.

Here we test whether collective decision-making based on quorum sensing is compatible with estimating the average quality of a fluctuating resource as predicted by homogenization theory. We did this by giving *T. albipennis* ant colonies a choice between a constant nest (CN) of mediocre quality and a fluctuating nest (FN) that is good 25%, 50% or 75% of the time and otherwise poor. Although nest sites in the field are unlikely to fluctuate relatively rapidly in quality in terms of light ingress, they may do so in terms of temperature^30^, for example, if the sun was repeatedly disappearing behind and reappearing between scudding clouds. Hence, we suggest that our experiments are not too divorced from the kinds of fluctuations that might actually occur in the natural world. In general, these ants may use light ingress to assess the integrity of a potential nest site^31^ (i.e. how many holes breach the walls of the cavity). Light-transmitting holes may allow enemies or inclement conditions to enter the nest. Thus high or fluctuating light levels may indicate a high-risk nest site. Choices between environments that are consistent or fluctuating in their risks and rewards have been the subject of great interest in behavioural ecology. Reviewing this substantial literature is beyond the scope of the present paper. However, we refer readers to two recent and lucid reviews by Kacelnik & El Mouden (2013)^32^ and by Fawcett *et al.* (2014)^33^.

In order to understand the possible mechanism by which the ants might be able to estimate the average quality of a fluctuating nest site, we also analyse a mathematical model modified from that in Pratt *et al.* (2002)^21^.

## Methods

**Figure 1:**
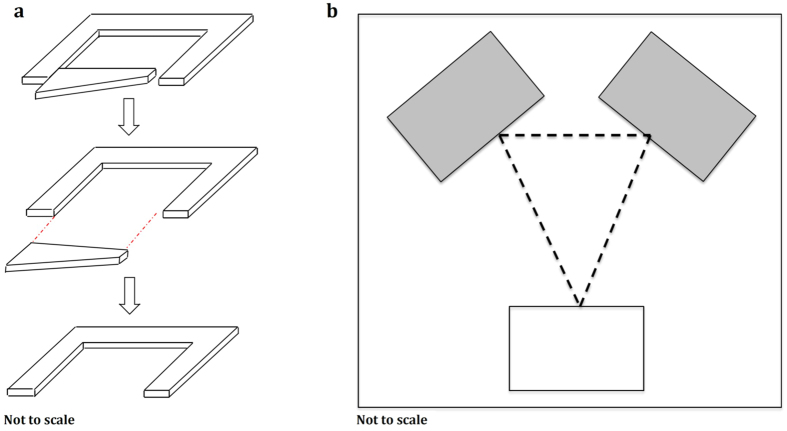
Experimental design: (**a**) A holding nest with one removable wall, the entrance width is 1 mm before wall removal and 60 mm after wall removal. (**b**) Experimental arena: the dark rectangles show the location of the two new nest sites (the Fluctuating Nest and the Constant Mediocre Nest) and the white rectangle represents the holding nest. The petri dish is 230 × 230 mm; the distance between the entrances of the two new nests is 90 mm; the distance between the entrance of the holding nest and the entrance of each new nest is 105 mm.

Thirty colonies were collected from the coast of Dorset on 21^st^ September 2013. Colonies had between 24 and 92 workers, one queen, and brood that were roughly as numerous as the workers within a colony^34^. The colonies were fed and housed according to standard protocols^7^. Prior to experimentation, all colonies were induced to emigrate into holding nests (Fig. 1a). Experiments began by placing each holding nest containing the test colony in the experimental arena and presenting it with two available target nests (Fig. 1b). Emigrations were induced by removing one of the holding nest’s walls to decrease its quality (Fig. 1a,b). Each colony had a choice between a nest that fluctuated in quality between good and poor (the fluctuating nest, FN) and a nest that was of constant mediocre quality (the constant nest, CN, Table 1). We made fluctuating nests change in quality by simply adding or removing a red filter from atop the upper microscope slide that formed the roof of the nest cavity. When such a red filter is in place, the nest cavity is perceived as dark by the ants, making it more attractive than when such a red filter is absent^18^. As a control for the minimal disturbance caused by the addition or removal of such a red filter from the fluctuating nest a clear filter was simultaneously added or removed from the constantly mediocre nest.

Fluctuating nests varied between being “good” and “poor” within each successive 10-min interval throughout the nest selection period. The proportion of time the fluctuating nest was either good or poor varied according to treatment type (Table 2). So for example, a fluctuating nest that was 25% good and 75% poor was good for 2.5 min and then poor for 7.5 min. Such a cycle was repeated until social carrying occurred to one of the new nest sites. To facilitate keeping track of the state in which each nest had to be within each 10-min interval over the 3-h observation period, fluctuating nests were always “good” first and “poor” second.

Each colony experienced two of the three treatments in a random order separated by a two-week rest period. The reason we did not use a fully balanced design with all colonies undergoing each of the three treatments was to reduce the numbers of emigrations each colony had to perform. This minimized total experimental time and any potential seasonal effects. We included Colony as a random factor in the statistical analyses to take full account of which colonies performed under which treatments.

We ran each replicate either until a quorum threshold was met in one of the new nests, indicated by social carrying to that site, or until 3 h had elapsed. We maintained direct observations throughout such 3-h periods. Every 10 min we directly counted and recorded the numbers of ants within each new nest site. We also recorded the occurrence of tandem runs; quorum thresholds, i.e. the number of ants in the new nest when active transport began; and the time elapsed from the start of the experiment until a quorum was attained.

### Statistical analysis

All statistical models were fitted with the Generalized Linear Mixed Model tool in SPSS 21 (Armonk, N. IBM SPSS Statistics for Windows. (2012)).

#### Nest site selection

Initial model: We fitted a multimodal (nominal) logistic regression mixed model with a logit link to all 60 emigrations. The response variable was the type of chosen nest with three possible values: Fluctuating, Mediocre, None. The predictors were: (a) the Ratio of good vs poor quality for the fluctuating nest with the three possible values: 25% G : 75% P, 50% G : 50% P and 75% G : 25% P as a fixed factor; (b) Colony size as a covariate; (c) interaction between the Ratio of good vs poor quality of the fluctuating nest and Colony size and (d) Colony ID with 30 possible values as a random factor. Neither the interaction nor Colony size on its own was statistically significant. When the model was run with just the Ratio of good vs poor quality for the fluctuating nest as a fixed factor predictor and Colony ID as the random factor, the likelihood of the ant colonies choosing the Fluctuating rather than the Mediocre nest was significantly higher when the Fluctuating nest was mostly of good quality (75% G : 25% P) than when it was mostly of poor quality (25% G : 75% P). However, only 63.3% of the predictions were correct (18.8% for No nest chosen, 75% for Fluctuating nest and 85% for Mediocre nest). For this reason we removed from the data the 16 emigrations in which the colonies made no nest choice.

Final model: We fitted a binary logistic regression mixed model with a logit link to the 44 emigrations by 27 colonies, in which the colony made a nest choice. The response variable was again the type of the chosen nest but this time it had only two possible values: Fluctuating and Mediocre. The predictors were again the Ratio of good versus poor quality for the fluctuating nest with the three possible values: 25% G : 75% P, 50% G : 50% P, 75% G : 25% P, as a fixed factor and Colony ID with 27 possible values, as a random factor (which was not significant, z = 0.597, p = 0.550). The model structure included colony ID as the subjects. As in the initial model, neither the interaction between the Ratio of good vs poor quality of the fluctuating nest and Colony size, nor Colony size on its own was statistically significant and both were removed. The results were qualitatively the same as the results from the initial model. However, the overall predictive accuracy of the final model was much higher, 81.8% overall (79.2% and 85.0% of the Fluctuating and Mediocre nest choices, respectively, were predicted correctly). The sequential Bonferroni method was used for post-hoc pair-wise comparisons.

#### Ant accumulation within the new nest sites

We fitted a log-linear mixed model with a Negative binomial error structure and a log link to the 44 emigrations in which the colonies made a choice with a response variable Number of ants in new nest (range 0 to 11 per 10 min). The predictors were: (a) the ratio of good versus poor quality for the fluctuating nest with the three possible values: 25% G : 75% P, 50% G : 50% P, 75% G : 25% P, as a fixed factor; (b) type of nest chosen with two possible values: Fluctuating and Mediocre, as a fixed factor; (c) Time (min) as a covariate (d) all three pair-wise interactions between the above three predictors; (e) the three-way interaction between all three above predictors and (f) Colony ID as a random factor (which was significant, z = 3.081, p = 0.002). The full model had a significant three-way interaction (Table S1) and this was important for the interpretation of the dynamics of ant numbers in the two new nests in relation to treatment. The full model had AIC of 4392.443. The Pearson residuals ranged from −1.581 to 7.755. Reducing the model by removing the three-way interaction and two of the two-way interactions made minimal improvement to the AIC (AIC = 4324.441) and a full model with a Poisson error structure and a log link had a worse fit to the data (AIC = 4583.925). Hence the full model with Negative binomial error structure and a log link was accepted as the final model.

#### Quorum thresholds

We fitted a log-linear mixed model with a Poisson error structure and a log link to the 44 emigrations in which the colonies made a choice with a response variable Quorum threshold (range: 4 to 18).

Full model: The predictors were: (a) the ratio of good versus poor quality for the fluctuating nest with the three possible values: 25% G : 75% P, 50% G : 50% P, 75% G : 25% P, as a fixed factor; (b) type of nest chosen with two possible values: Fluctuating and Mediocre, as a fixed factor; (c) the interaction between the above two fixed factors; (d) Colony size as a covariate; (e) Time to achieve quorum (min) as a covariate and (f) Colony ID as a random factor. This full model had AIC of 61.718 and only Colony size had a significant effect at the 5% significance level. The final model was selected on the basis of minimizing AIC from a group of hierarchical models based on the full model.

The final model had AIC of 45.308 and included only Colony size and the random factor Colony ID (the effect of which could not be estimated due to the poor fit of the model) as predictors. The Pearson residuals ranged from −1.551 to 2.718. The effect of Colony size was significant at the 5% significance level.

We also fitted a linear mixed model to the 44 emigrations in which the colonies made a choice with a response variable Time to achieve quorum (min). The full model contained the same predictors as the model for Quorum threshold above except that this time Quorum threshold was a covariate (the effect of the random factor Colony ID was not significant, z = 0.773, p = 0.440). This full model was also the final model because its AIC of 409.778 was the lowest among a group of hierarchical models based on the full model. None of the predictors in this model had a significant effect on Time to achieve quorum. The model residuals were compatible with a normal distribution (Shapiro-Wilk test statistic = 0.960, d.f. = 44, p = 0.127).

## Results

### Nest site selection

The colonies achieved a quorum threshold, in one of the new nest sites, within the 3-h observation period, in 44 of the 60 experimental replicates. Failures to achieve a quorum were evenly distributed among the three treatments. The following analyses focus entirely on the 44 cases in which colonies were quorate in a new nest and instigated an emigration.

**Figure 2:**
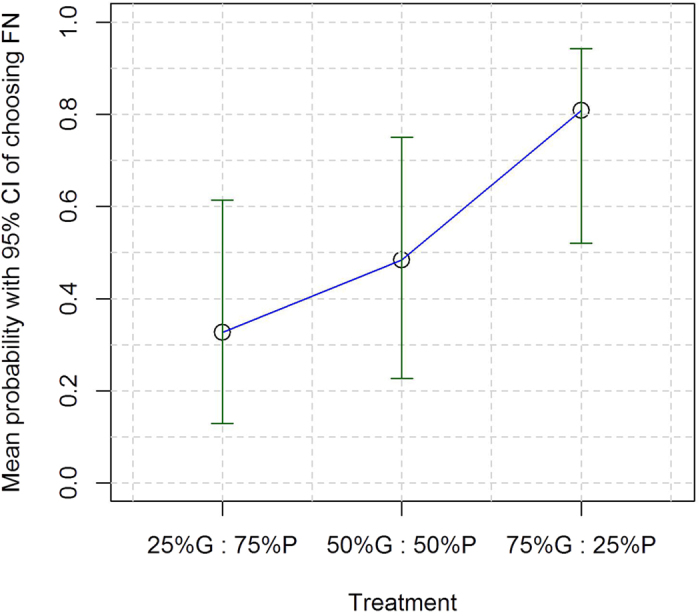
The mean probability with 95% CIs of a *T*. *albipennis* colony choosing the fluctuating nest (FN) instead of the constant nest (CN) of mediocre quality in relation to the percentage of time it was good vs poor quality; 25% G : 75% P is 25% good : 75% poor quality, 50% G : 50% P is 50% good : 50% poor quality, 75% G : 25% P is 75% good : 25% poor quality.

Ant colonies chose the Fluctuating rather than the Mediocre nest with an average probability of 0.809 when the Fluctuating nest was mostly good (75% G : 25% P); with an average probability of 0.484 when it was good and poor for identical periods (50% G : 50% P) and with an average probability of 0.327 when it was mostly poor (25% G : 75% P). The difference between the highest and lowest of these probabilities is statistically significant (GLMM with Bonferroni post-hoc tests, 75% G vs 25% G: t = 2.972, d.f. = 41, p = 0.015; 75% G : vs 50% G: t = 1.856, d.f. = 41, p = 0.141; 50% G vs 25% G: t = 0.833, d.f. = 41, p = 0.410; Fig. 2).

### Tandem runs

In general there were few tandem runs. Tandem runs rarely occurred in these experiments as the independent discovery rate of nest sites was probably high, since the new nest sites were close to the old nest, and the ants used quorum thresholds based on a small number of their nestmates^8^,^28^. Among the 44 replicates in which colonies initiated an emigration, the mean number of tandem runs was less than 1 (N = 44, mean = 0.909, s.d = 1.537, median = 0, Q1 = 0, Q3 = 2).

### Ant accumulation within the new nest sites

**Figure 3:**
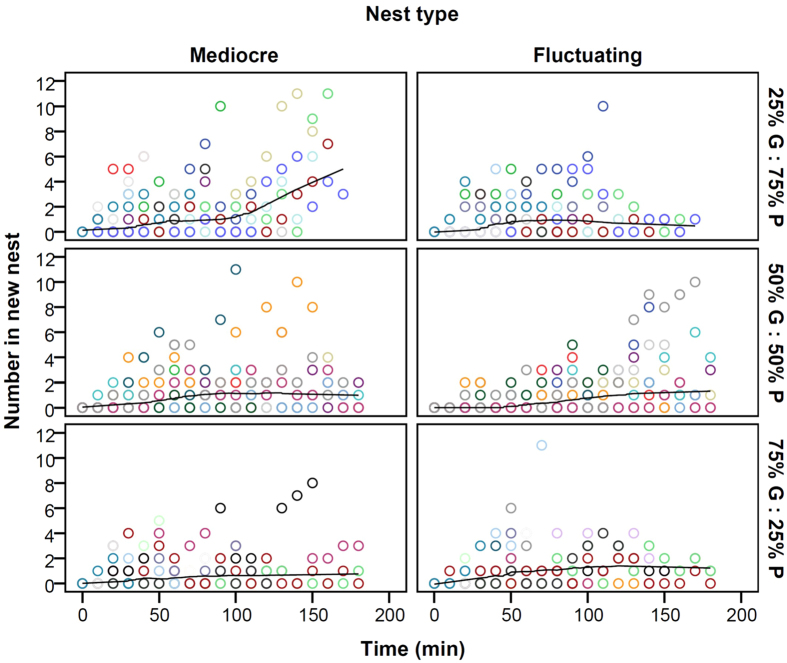
The numbers of ants within each of the nests, constant of mediocre quality and fluctuating, through time for each of the three treatments. Treatment was the percentage of time the fluctuating nest was good vs poor quality; label meaning is as in caption to Fig. 2. The different colours represent the 27 different colonies, which made a nest choice during 44 out of a total of 60 emigrations of 30 colonies. Seventeen colonies are represented twice because they made a nest choice in both of the treatments they underwent. To guide the eye, the points for each combination of nest type and treatment over time are fitted by a Loess curve with an Epanechnikov kernel.

On average the number of ants increased significantly over time both in the Mediocre nest and in each of the three types of Fluctuating nest (the time coefficient was significantly different from 0, with t-values in the range of 5.850 to 11.876, d.f. = 1152 and p < 0.001 in each case; Fig. 3). When the Fluctuating nest was 25% G : 75% P, the accumulation rate in the Mediocre nest was significantly higher than in the Fluctuating nest (time coefficients 0.022 and 0.013, respectively, t = 3.728, d.f. = 1152, p < 0.001, Fig. 3). By contrast, when the Fluctuating nest was 50% G : 50% P, the accumulation rate in the Mediocre nest was significantly lower than in the Fluctuating nest (time coefficients 0.010 and 0.019, respectively, t = −4.218, d.f. = 1152, p < 0.001, Fig. 3). The accumulation rate in the Mediocre nest was also lower than in the Fluctuating nest when the latter was 75% G : 25% P but this difference was not statistically significant (time coefficients 0.013 and 0.014, respectively, t = −0.337, d.f. = 1152, p = 0.736, Fig. 3). There was no significant difference in the accumulation rate in the Mediocre nest when the Fluctuating nest was 50% G : 50% P compared to when it was 75% G : 25% P but the accumulation rates in the Fluctuating nest were significantly different (t = −1.364, p = 0.173 and t = 2.543, p = 0.011, respectively, d.f. = 1152 in both cases; Fig. 3).

### Quorum thresholds

**Figure 4:**
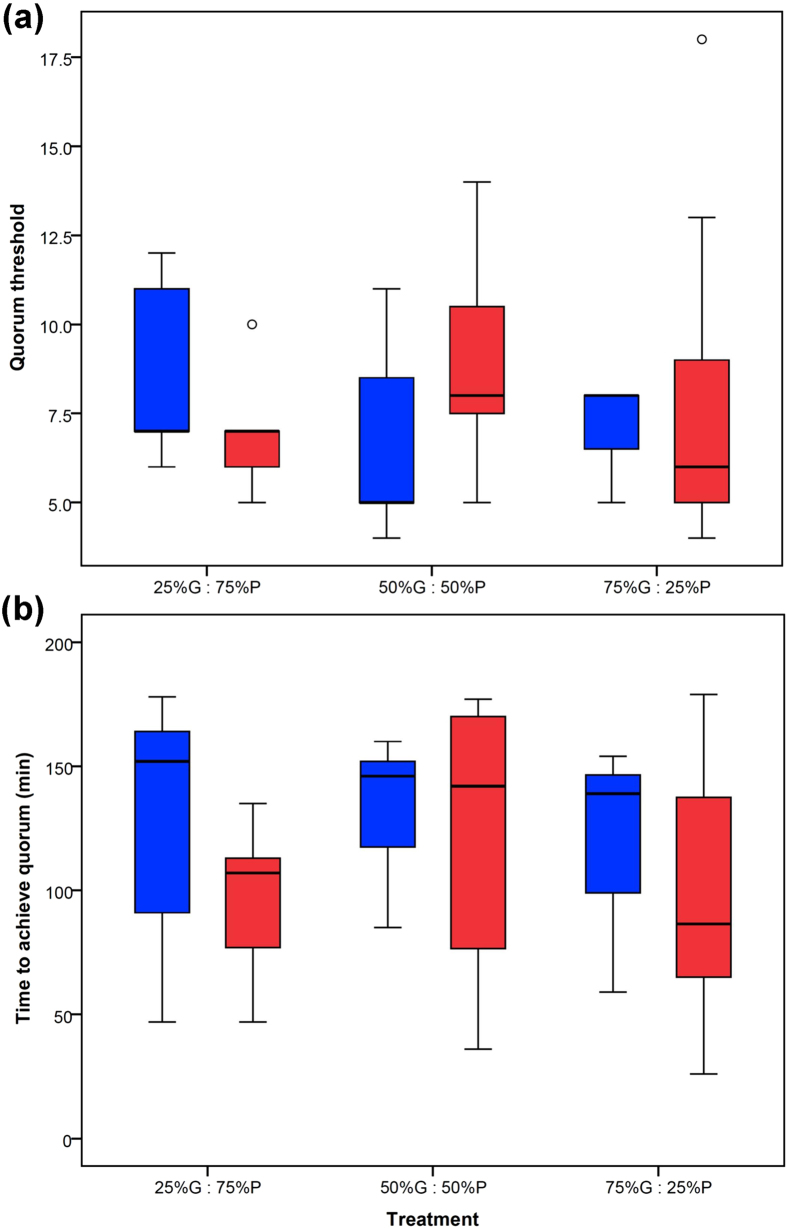
Quorum thresholds for the 44 emigrations for which 27 colonies made a nest choice: (**a**) the number of ants in the quorum and (**b**) the time taken to achieve a quorum threshold within a new nest site for the three treatments based on the percentage of time the fluctuating nest was good versus poor quality. The treatment labels are as in the caption to Fig. 2. Blue denotes the Constant Nest and red the Fluctuating Nest. 25% G : 75% P: N_CN_ = 10, N_FN_ = 5; 50% G : 50% P: N_CN_ = 7, N_FN_ = 7; 75% G : 25% P: N_CN_ = 3, N_FN_ = 12; the box of each boxplot encompasses the inter-quartile range (IQR); a line within the box depicts the median (which can coincide with one of the upper or lower quartiles); whiskers extend to the furthest value within a range of 1.5 times the IQR; any values beyond 1.5 times the IQR are defined as outliers and plotted as a o.

Quorum threshold was not significantly affected by the interaction between the ratio of good versus poor quality for the fluctuating nest and the type of nest chosen (full GLMM, F_2,36_ = 1.335, p = 0.276, Fig. 4a), and neither factor had a significant effect on its own (full GLMM, F_2,36_ = 0.007, p = 0.993 and F_1,36_ = 0.338, p = 0.565, respectively). The covariate Time to achieve quorum (min) did not have a significant effect on quorum threshold either (full GLMM, F_1,36_ = 0.058, p = 0.810). However, Colony size had a significant effect on Quorum threshold (full GLMM, F_1,36_ = 8.032, p = 0.007; final GLMM, F_1,42_ = 9.018, p = 0.004). Larger colony sizes were associated with larger quorum thresholds (Fig. S1). Such a relationship has been shown before^34^.

Time to achieve quorum (min) was not significantly affected by the interaction between the ratio of good versus poor quality for the fluctuating nest and the type of nest chosen (LMM, F_2,36_ = 0.142, p = 0.868, Fig. 4b), and neither factor had a significant effect on its own (LMM, F_2,36_ = 0.393, p = 0.678 and LMM, F_1,36_ = 1.562, p = 0.219, respectively). In addition, neither the covariate Colony size (LMM, F_1,36_ = 0.950, p = 0.336) nor Quorum threshold (LMM, F_1,36_ = 0.093, p = 0.763) had a significant effect on Time to achieve quorum.

### Mathematical Modelling

**Figure 5:**
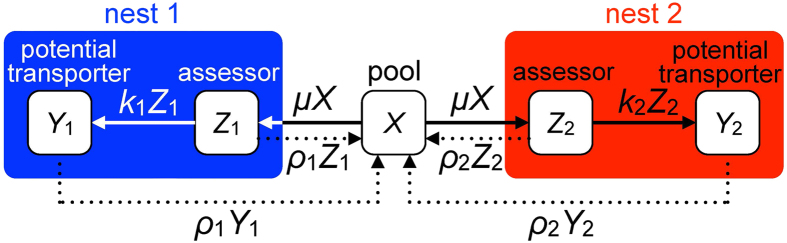
Flow chart of the mathematical model. For more details, please see the text.

The model is that of Pratt *et al.* (2002)^21^, modified to account for the different experimental set-up. The possible states of the active ants and the fluxes between these states are shown in Fig. 5. Whilst passive ants or brood items remain at the original nest, the dynamics are given by:
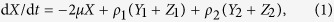

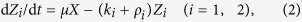

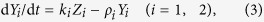

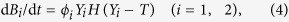
where *X* is the number of active ants that have not yet discovered a new nest, *Z*_*i*_ is the number of ants assessing nest *i* (*i* = 1, 2), *Y*_*i*_ is the number of ants that are potential transporters to nest *i*, in that they have made a positive assessment of it and will transport to it when the quorum threshold has been attained, and *B*_*i*_ is the numbers of passive ants and brood items in new nest *i*. The parameter *T* is the quorum threshold, so that when the number *Y*_*i*_ of potential transporters exceeds *T* they will begin to transport to nest *i*, at a per capita rate *ϕ*_*i*_. The function H is the Heaviside step function, so that H(*Y*_*i*_ − *T*) = 0 when *Y*_*i*_ < *T* and H(*Y*_*i*_ − *T*) = 1 when *Y*_*i*_ > *T*. The parameter *μ* is the per capita rate of discovery of each new nest. The parameter *k*_*i*_ is the per capita rate at which assessors of nest *i* become potential transporters to it. The parameter *ρ*_*i*_ is the per capita rate at which assessors of or potential transporters to nest *i* abandon it and return to the pool *X* of active ants, from where they may become assessors of and then potential transporters to the other nest. This is an indirect process of switching, in contrast to the direct switching process assumed by Pratt *et al.* (2002)^21^, and is one of the differences between our model and theirs. There are two reasons why we prefer indirect switching here. First, tandem running was very rare in these experiments suggesting that recruitment prior to the attainment of quorum thresholds is negligible and that here scouts discover the new nest sites independently. Second, direct switching was based in part on the notion that individual ants actively seek to compare different nest sites. This is now in question^35^,^36^. Hence, here we favour the simpler indirect switching hypothesis. The other difference is that, in accordance with the experimental observations, no tandem running is incorporated into our model. Note that, if nest 1 is the CN and nest 2 the FN, then *k*_1_ and *ρ*_1_ will be constant, while *k*_2_ and *ρ*_2_ will vary following the variations in quality of the FN, taking high and low values (low abandonment rate), respectively, when the nest quality is high, and low and high values, respectively, when the nest quality is low.

Values of the constant rate parameters are *μ* = 0.03, *k*_*1*_ = 0.020, *ρ*_1_ = 0.008, all per minute. Parameters *k*_2_ and *ρ*_2_ alternate between (*k*_2_, *ρ*_2_) = (0.024, 0.004), corresponding to the good phase of the FN, and (*k*_2_, *ρ*_2_) = (0.016, 0.012), corresponding to the poor phase of the FN, unless otherwise stated.

**Figure 6:**
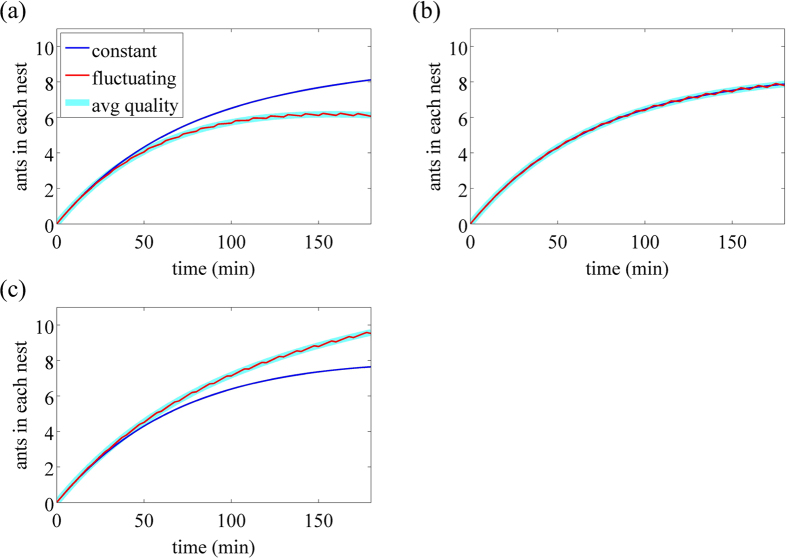
Time courses of the number of ants in each nest for (**a**) 25% G : 75% P, (**b**) 50% G : 50% P, (**c**) 75% G : 25% P. One of the advantages of the mathematical modelling is that we can run the model for exactly the average properties of the FN. Hence we can plot the results for such a constant-quality nest, which we call Average Nest (AN), the quality parameters of which are set to the running average of those for the FN. The blue and red lines represent the number of ants in the CN and FN, respectively. The cyan lines represent the number of ants in the AN when the colony is presented with the AN and CN. The time courses for the CN under this condition are omitted because they almost completely overlap with those for the CN when the FN and CN are presented (i.e., blue lines). In (**b**), all three lines overlap heavily with each other.

The quorum threshold *T* is taken to be 7 ants, and *ϕ*_1_ = *ϕ*_2_ = 0.099 per minute. The values for *μ* and *T* are from the current experiments (or very similar ones, Stuttard personal observations), but note that *T* in Pratt *et al.* (2002)^21^ (and hence in this model) represents the number of potential transporters *Y* for a quorum. The total number of ants, *Y*_*i*_ + *Z*_*i*_, has to be higher than *T* for a quorum to be reached at nest *i*. This is why quorum is not reached in the numerical simulations (Fig. 6 and Figs S2, S3 and S4) even when ant populations at a given nest are somewhat higher than 7. The values for *ϕ*_1_ and *ϕ*_2_ are from Pratt *et al.* (2002)^21^. Those for *k*_1_ and *k*_2_ are guided by Pratt *et al.* (2002)^21^, who took *k*_1_ = 0.020 and *k*_2_ = 0.016 for a site 2 that was clearly better than site 1, and *k*_1_ = 0.020 and *k*_2_ = 0.019 if the difference in quality was smaller. The values of *ρ*_1_ and *ρ*_2_ are also guided by Pratt *et al.* (2002)^21^, who had *direct* transfer of allegiance to a better site at rate *ρ*_12_ = 0.008 for a clearly better site and *ρ*_12_ = 0.004 for a marginally better one. These parameters are not directly comparable to ours, because we have indirect transfer of allegiance, but they give an idea of order of magnitude. Note typical time scales for the *k*s, 1/0.016 = 62.5 min to assess a poor nest positively, 1/0.020 = 50 min for a mediocre nest, 1/0.024 = 42 min for a good nest, and for the *ρ*s, 1/0.012 = 83 min to abandon a poor nest, 1/0.008 = 125 min for a mediocre nest, 1/0.004 = 250 min for a good nest. The values of the *k*s and *ρ*s may be used to estimate the probability *p* that an ant that discovers a new nest eventually assesses it positively rather than abandoning it, *p* = *k*/(*k* + *ρ*), or alternatively these probabilities (if known empirically) and the *k* values may be used to estimate the *ρ*s. The probabilities given the parameter values above are approximately 57%, 71% and 86% that a poor, a mediocre and a good nest are eventually assessed positively by a given discoverer, respectively.

### Model Results

We implemented the model using the ode45 solver of MATLAB. Because the solver is not guaranteed to work out discontinuity in the differential equation, we segmented the time domain into windows in each of which the nest quality is constant for the FN. For example, for the FN in the case of 25% G : 75% P, we first used the ode45 to integrate the model between *t* = 0 and *t* = 2.5 by assuming the constant parameter values representing the good quality. Then, using the state variables at *t* = 2.5 as the initial condition, we integrated the model till *t* = 10 with the parameter values representing the poor quality, completing the second time window, and so on.

Time courses of the number of ants in each nest, defined as *Z*_*i*_ + *Y*_*i*_ + *B*_*i*_ (*i* = 1, 2) are shown by the CN and FN by the blue and red lines, respectively, in Fig. 6. In Fig. 6(a), the FN is of poor quality 75% of the time, corresponding to 25% G : 75% P. The FN started as the good nest at *t* = 0, switched to the poor nest at *t* = 2.5, then switched back to the good nest at *t* = 10 and so forth. Fig. 6(a) shows that the number of ants grows faster in the CN than FN, which is consistent with the experimental results (Figs 2 and 3). The time course for the FN looks smooth. If we magnify the figure, we will see that the time course consists of a concatenation of piecewise smooth trajectories having different accumulation rates corresponding to the good and poor nest qualities.

To show that the model ant colony is good at calculating the running average of the nest quality, we carried out additional numerical simulations in which ants are presented with two constant nests. The first nest is the same as the usual CN. The other constant nest, which we call the averaged nest (AN), has the quality parameters equal to the running average of those for the FN. In other words, we set *k*_2_ = 0.25 × 0.024 + 0.75 × 0.016 = 0.018 and *ρ*_2_ = 0.25 × 0.004 + 0.75 × 0.012 = 0.006 for the AN throughout the dynamics. The number of ants in the AN when the colony is presented with the CN and AN are shown by the thick cyan line in Fig. 6(a). The number of the ants in the CN is not shown in this and the following figures because the time course almost completely overlaps with that for the CN when the CN and FN are presented (blue line). The figure shows that the time course for the AN heavily overlaps with that for the FN except that the latter accompanies slight fluctuations owing to the fluctuating nest quality. In other words, the colony subjected to the FN behaves as if the FN were a constant nest whose quality were the running average of that of the FN.

The time courses when the colony is presented with the CN and FN in 50% G : 50% P (i.e., 50% good and 50% poor quality for the FN) are shown in Fig. 6(b). The two time courses are hardly distinguishable. This result is consistent with the experimental results, in which the colonies did not differentiate between the two nests under this treatment (Fig. 2) even though the dynamics of ant accumulation within the nest sites showed a significantly higher accumulation rate in the FN (Fig. 3). If we replace the FN by the AN, the time course of the number of ants in the AN behaves almost the same (cyan line in Fig. 6(b)). Finally, the time courses for 75% G : 25% P (i.e., 75% good and 25% poor) are shown in Fig. 6(c). The figure shows that FN is favoured over the CN, consistent with the experimental results (Fig. 2) even though the higher population accumulation rate in the FN was not statistically significantly different (Fig. 3). Replacement of the FN by the AN does not change the results (cyan line in Fig. 6(c)). In sum, the model colony is good at calculating the running average of the quality under the three treatments.

The calculation of the running average might be easy in Fig. 6 because the fluctuation in the parameter values (i.e., *k*_2_ and *ρ*_2_) for the FN was mild. To address this point, we carried out the same set of numerical simulations with the amplitude of the switching parameters magnified close to the possible maximum values. In other words, we set (*k*_2_, *ρ*_2_) = (0.038, 0.001) for the good phase of the FN and (*k*_2_, *ρ*_2_) = (0.002, 0.015) for the poor phase of the FN. The parameters for the CN were unchanged, i.e., (*k*_1_, *ρ*_1_) = (0.020, 0.008). The numerical results for the three treatments are shown in Fig. S2. Despite the larger fluctuations for the results for the FN (red lines), the model colony is still good at approximating the running average of the nest quality (cyan lines) in each treatment. The results were also robust if we increased the period of the nest fluctuation to 30 min, which was adverse to the calculation of the running average (Figs S3 and S4).

It should be noted that, in Fig. 6, the quorum was not reached within 180 min in any case with the current choice of the parameter values. However, we believe that the results would be qualitatively the same if we changed the parameter values to facilitate quorum attainment and the rapid transport of passive ants and brood items that ensues. In fact, we observed qualitatively the same results with quorum attainment for some parameter values (Figs S2(c) and S4(c)).

## Discussion

The ants’ algorithm seems to be effective in estimating the average quality of a new nest site even when the quality of that nest site fluctuates through the assessment period. The key to this extraordinary ability almost certainly lies with the ants’ use of quorum sensing whereby they only commit to accepting and beginning an emigration to a new nest when a certain abundance of their nest mates have accumulated within such a nest site^21^,^26^. One likely explanation is that individual ants simply spend time within a nest site as a function of its quality (Fig. 3). Hence, better nest sites retain more ants and are more likely to achieve a quorum threshold faster than poorer alternatives. This very simple mechanism would represent a powerful algorithm for calculating the running average of a nest site’s quality. Thus a fluctuating nest may capture and retain the attention and presence of individual ants when it is of high quality whilst causing such ants slowly to drift away when it is of low quality. When a fluctuating nest is good for 25% of the time and poor for 75% of the time it retains the attention of fewer ants than a constantly mediocre nest. Indeed, in such a situation the ants tend to prefer the mediocre alternative (Figs 2,3). Conversely, when a nest is good for 75% of the time and poor for 25% of the time it is better able to retain the attention of visiting ants than a mediocre nest and colonies are more likely to choose the former (Figs 2,3). When a nest fluctuates between equal periods of being excellent or poor, overall it should retain as many visiting ants as a constantly mediocre nest, and indeed, colonies tend to show no preference between such alternatives (Fig. 2) even though the dynamics of ant accumulation within the new nests favoured the fluctuating nest (Fig. 3). Such a difference between the final choice and the dynamics might not be as unexpected as it first appears. It is remarkable that the final choices are compatible with the theory given the deterministic nature of the model and the variable behaviour of individual scouts. This is probably resolved by the powerful averaging that quorum sensing provides.

Our mathematical modelling, based on a simplification of that in Pratt *et al.* (2002)^21^, suggests that we have captured some understanding of the algorithms and dynamics of the decision-making procedures. In our experiments, we found no significant dependency of the quorum threshold, or the time to achieve a quorum threshold, on nest qualities be they constant or fluctuating. This was probably because quorum thresholds seem to be largely set by environmental conditions including the condition of the original nest site^7^,^25^ and in these experiments these factors were constant.

Our extensive experience with the model system of collective decision-making in *Temnothorax albipennis*^7^,^8^,^17^,^18^,^19^,^20^,^21^,^37^ facilitated our design of experiments in which the nest that fluctuated between being good and poor for equal periods of time was deemed similar by the ants to a constantly mediocre nest site (Fig. 2). Indeed, our overall design was such that, pooled over all replicates and treatments, colonies were exposed to new nests be they fluctuating or constant that were ***on average*** extremely similar. That is, the first and third treatments, 25% G : 75% P plus 75% G : 25% P, should average to 50% G : 50% P, namely ((25% Good : 75% Poor) + (75% Good : 25% Poor))/2 should roughly equal (50% Good : 50% Poor). Indeed, when we pool the results of all of the replicates, we find that overall colonies choose the fluctuating nest 24 times and the constantly mediocre nest 20 times. This small difference is not significant (Binomial Test, p = 0.326). All this suggests that the ants’ quorum-based algorithm is remarkably reliable and robust.

Some of the parameters in the mathematical model are constant, while others vary on a time scale that is fast (min) compared to that of the experiment as a whole (h). Numerical results show that the solution changes very little if the variable parameters are replaced by their average values (Fig. 6). This is an application of the mathematical theory of homogenisation^16^. The appropriate average value to use depends on the application, but here it may be shown that, before the quorum threshold is reached and recruitment begins, the appropriate average is the mean value over a period.

In our numerical simulations, the behaviour of ants in response to the FN was approximately equal to that in response to the constant nest whose parameter values were the temporal averages of those for the FN (Fig. 6). The present experimental results also support this finding although, in quantitative terms, the average of the good and the poor nests may not be equal to the mediocre nest in terms of the quality. Ant colonies seem to be good at calculating the running average of the nest quality. Mathematically, application of the homogenization theory to our system requires that the fluctuation of the FN must be fast enough. In fact, in our experiments and numerical simulations, the switching occurred relatively slowly such that there were not many cycles contained in the entire observation time (18 cycles with a period of 10 min in the experiments and the numerical results shown in Fig. 6 an S2, and six cycles with a period of 30 min in the numerical results shown in Figs S3 and S4). Therefore, albeit without mathematical guarantees, the implication is that homogenization theory may extend to the case of relatively slow fluctuations in various real systems including the present one.

We suggest that the experiments we have conducted here provide new and important insights into the benefits of quorum sensing: namely, the ability to produce a very accurate corporate assessment over a relatively prolonged period. The ants by almost literally voting with their feet provide appropriate collective assessments over time periods that are neither too short to be representative of a meaningful average nor too long to place their colony in undue danger.

## Additional Information

**How to cite this article**: Franks, N. R. *et al.* How ants use quorum sensing to estimate the average quality of a fluctuating resource. *Sci. Rep.* **5**, 11890; doi: 10.1038/srep11890 (2015).

## Supplementary Material

Supplementary Information

## Figures and Tables

**Table 1:** Attributes of the experimental nests. All nests had an internal cavity of 55 × 30 mm.

**Nest type**	**Attributes**		
	**Light level**	**Height (mm)**	**Entrance width (mm)**
Good	Dark	1.6	4
Mediocre	Bright	1.6	1
Poor	Bright	1.6	4

**Table 2:** Description of each treatment. Light levels were manipulated by adding and removing a red filter atop the fluctuating nest.

**Treatment**	**Procedure for every 10-min interval**
1) 25% Good : 75% Poor	Dark for 2.5 min and bright for 7.5 min
2) 50% Good : 50% Poor	Dark for 5 min and bright for 5 min
3) 75% Good : 25% Poor	Dark for 7.5 min and bright for 2.5 min
