# Supplementary material for: How ants use quorum sensing to estimate the average quality of a fluctuating resource
Source: Sci Rep. 2015 Jul 8;5:11890. doi: 10.1038/srep11890 (PMC4495386; doi:10.1038/srep11890)
Supplement: Supplementary Information [file srep11890-s1.pdf]

How ants use quorum sensing to estimate the average quality of a fluctuating resource.

Nigel R. Franks, Jonathan P. Stuttard, Carolina Doran, Julian C. Esposito, Maximillian C. Master, Ana B. Sendova-Franks, Naoki Masuda, Nicholas F. Britton

Supplementary Information

Table S1. Fixed effects for the log-linear mixed model with a Negative binomial error structure and a log link applied to the 44 emigrations in which the colonies made a choice with a response variable Number of ants in new nest. Nest type is Fluctuating or Mediocre. Treatment is the percentage of time the Fluctuating nest was good vs poor quality: 75% G : 25% P, 50% G : 50% P, 25% G : 75% P.

Fixed Effects

Target:No. in new nest

Source	F	df1	df2	Sig.
Corrected Model ▼	38.056	11	1,152	.000
Treatment	1.833	2	1,152	.160
Nest_type	3.043	1	1,152	.081
Time_min	323.020	1	1,152	.000
Nest_type*Treatment	10.619	2	1,152	.000
Time_min*Treatment	1.883	2	1,152	.153
Time_min*Nest_type	0.017	1	1,152	.896
Time_min*Nest_type*Treatment	15.550	2	1,152	.000

Probability distribution:Negative binomial
Link function:Log

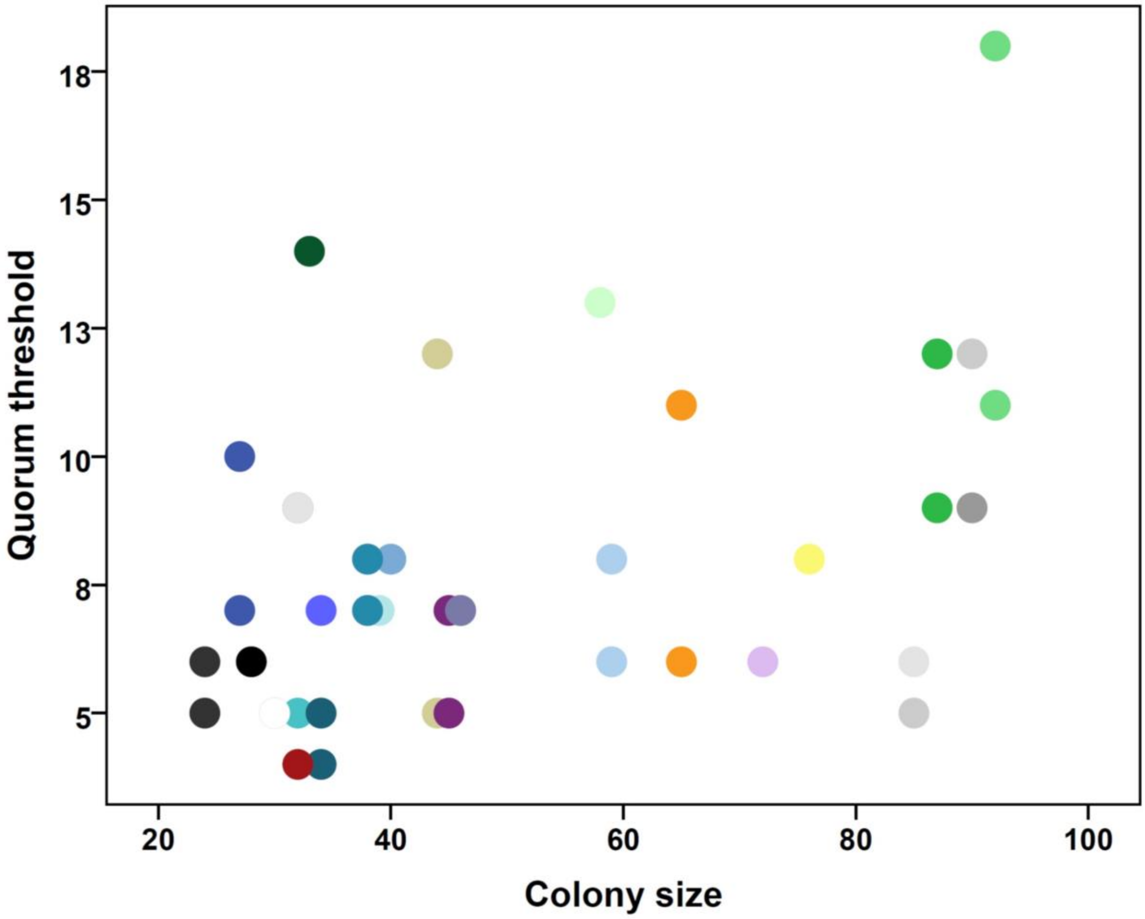

Figure S1. Positive relationship between quorum threshold and colony size for the 44 emigrations by 27 colonies (out of a total of 30), in which the colony made a nest choice. Different colours represent different colonies. Seventeen colonies are represented twice because they made a nest choice in both of the treatments they underwent. There are seven overlapping values. Hence 37 points are visible in the plot.

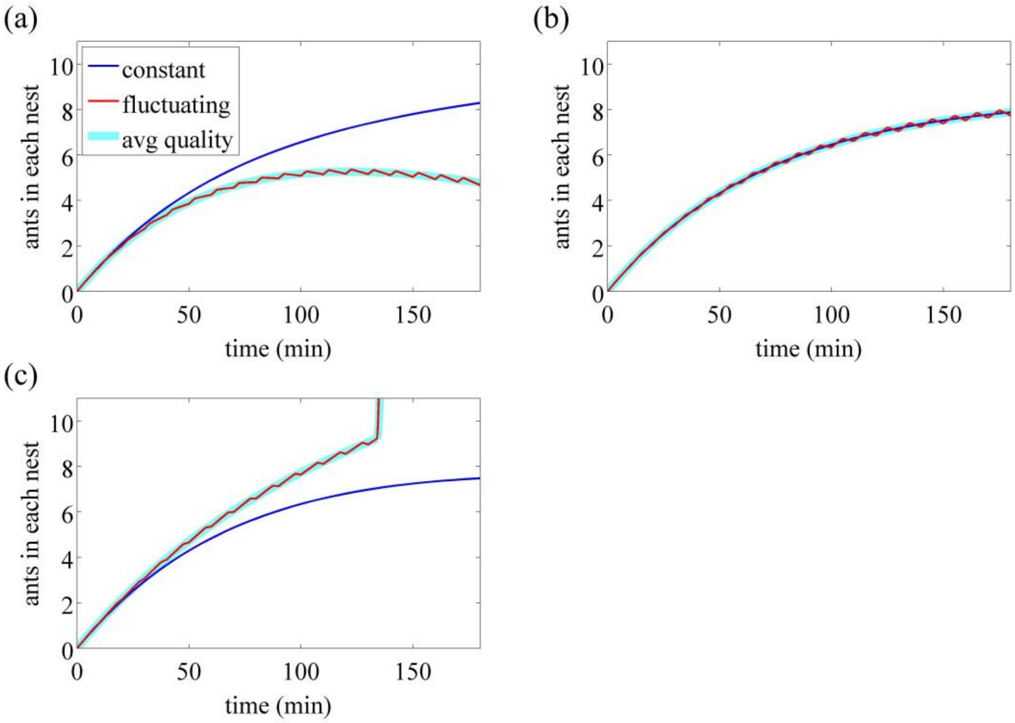

Figure S2. Time courses of the number of ants in each nest when the fluctuation of the FN has a larger amplitude than in the case of Fig. 6. (a) 25% G : 75% P, (b) 50% G : 50% P, (c) 75% G : 25% P. We set $(k_2, \rho_2) = (0.038, 0.001)$ and $(k_2, \rho_2) = (0.002, 0.015)$ for the good and poor phases of the FN, respectively. In (c), the steep increase represents rapid influx of passive workers and brood items that are carried into the nest site after quorum attainment.

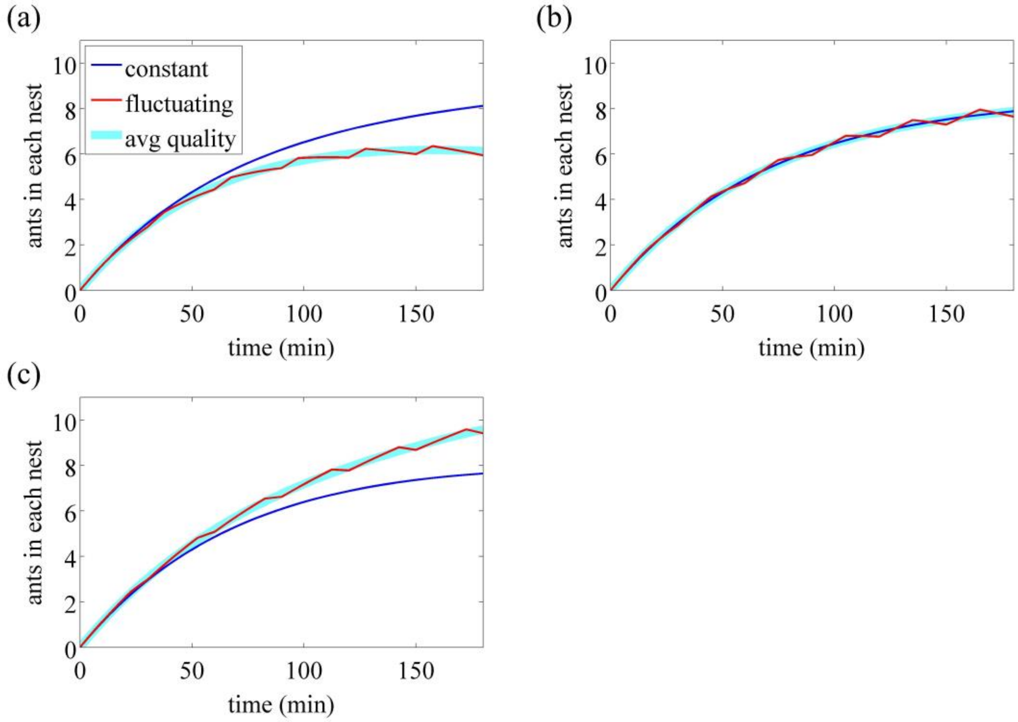

Figure S3. Same as Fig. 6, but with a period of 30 min for the FN. (a) 25% G : 75% P, (b) 50% G : 50% P, (c) 75% G : 25% P.

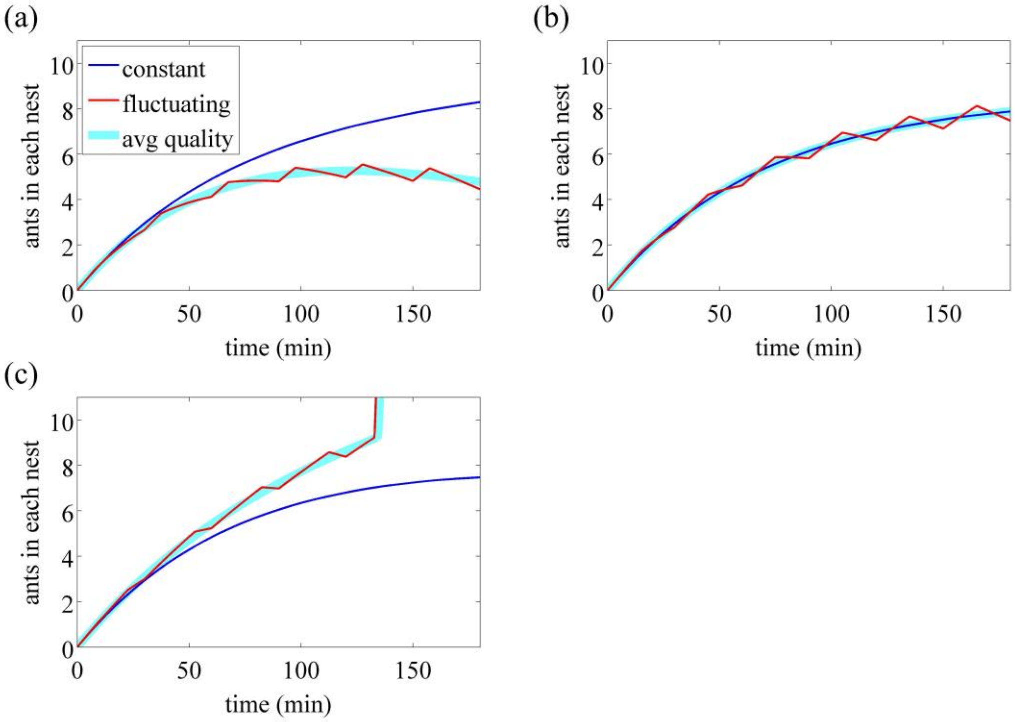

Figure S4. Same as Fig. S2, but with a period of 30 min for the FN. (a) 25% G : 75% P, (b) 50% G : 50% P, (c) 75% G : 25% P. In (c), the steep increase represents rapid influx of passive workers and brood items that are carried into the nest site after quorum attainment.
